# Anticancer Activity, Antioxidant Activity, and Phenolic and Flavonoids Content of Wild* Tragopogon porrifolius* Plant Extracts

**DOI:** 10.1155/2016/9612490

**Published:** 2016-11-24

**Authors:** Fuad Al-Rimawi, Suzi Rishmawi, Sharehan H. Ariqat, Mahmoud F. Khalid, Ismail Warad, Zaidoun Salah

**Affiliations:** ^1^Chemistry Department, Faculty of Science and Technology, Al-Quds University, P.O. Box 20002, Jerusalem, State of Palestine; ^2^Al-Quds-Bard College, Al-Quds University, Abu Dies, East Jerusalem, State of Palestine; ^3^Department of Chemistry, Science College, An-Najah National University, P.O. Box 7, Nablus, State of Palestine

## Abstract

*Tragopogon porrifolius*, commonly referred to as white salsify, is an edible herb used in folk medicine to treat cancer. Samples of* Tragopogon porrifolius* plant grown wild in Palestine were extracted with different solvents: water, 80% ethanol, and 100% ethanol. The extracts were analyzed for their total phenolic content (TPC), total flavonoid content (TFC), and antioxidant activity (AA). Four different antioxidant assays were used to evaluate AA of the extracts: two measures the reducing power of the extracts (ferric reducing antioxidant power (FRAP) and cupric reducing antioxidant power (CUPRAC)), while two other assays measure the scavenging ability of the extracts (2,2-azino-di-(3-ethylbenzothialozine-sulphonic acid (ABTS)) and 2,2-diphenyl-1-picrylhydrazyl (DPPH)). Anticancer activity of the plant extracts were also tested on HOS and KHOS osteosarcoma cell lines. The results revealed that the polarity of the extraction solvent affects the TPC, TFC, and AA. It was found that both TPC and AA are highest for plant extracted with 80% ethanol, followed by water, and finally with 100% ethanol. TFC however was the highest in the following order: 80% ethanol > 100% ethanol > water. The plant extracts showed anticancer activities against KHOS cancer cell lines; they reduced total cell count and induced cell death in a drastic manner.

## 1. Introduction

*Tragopogon porrifolius,* commonly known as salsify, oyster plant, and vegetable oyster belonging to Asteraceae family, is one type of* Tragopogon* species.* Tragopogon porrifolius* is grown in the wild and cultivated in the Mediterranean regions [1]. Every part of the plant including the roots, leafy shoots, and open flowers are edible [1]. The nutritional value of this plant has been attributed to its monounsaturated and essential fatty acids, vitamins, and polyphenols [2, 3]. It has been recorded since ancient times for traditional medicine. It is currently widely spread over the Middle Easter area, Europe, and North America [4].

*Tragopogon porrifolius* is a vegetable that is high in antioxidants which help in the process of preventing diseases by eliminating peroxides and free radicals from diets [5]. Phenolic compounds, which are plant secondary metabolites, are well-known antioxidants and so play important roles in disease resistance [6, 7].

Antioxidant enzymes constitute the first line of defense against oxidative stress and damage caused by free radicals. When there is an imbalance between oxidative stress and antioxidant enzymes then there is a chance that diseases such as cancer, autoimmune disorders, aging, cardiovascular, and neurodegenerative diseases may develop [8, 9]. The body will defend itself by synthesizing antioxidants or intake of food or supplements that contain antioxidants such as* Tragopogon porrifolius* [10].

Until now, the scientific literature does not report about the antioxidant activity and phenolic content or flavonoid content of* Tragopogon* plant from Palestine. Abundant literature dealing with total phenolic content and antioxidant activity was published from different countries including those of the Middle East. Tenkerian et al. 2015 studied TPC, TFC, AA, and hepatoprotective and anticancer activities in vitro and in vivo of methanolic extract of* Tragopogon porrifolius* from Lebanon. Also, the extract of the aerial part of the plant was tested on rats with normal and damaged liver. Results were progressive on both rats on the levels of the liver antioxidants enzymes [1]. Mojarrab et al. 2014 [11] studied antioxidant activity and TPC of hydroethanolic extract of* Tragopogon buphthalmoides* from Iran. Asadi-Samani et al. 2015 studied medicinal plants with hepatoprotective activity in Iranian folk medicine and including* Tragopogon porrifolius *[12].

Investigation of the effects of water extract of* Tragopogon porrifolius *shoot on inflammation, oxidative stress, and hepatotoxicity using a rat model showed that after one month of* Tragopogon porrifolius *water extract intake, a significant decrease in the levels of serum cholesterol, triglyceride, glucose, and liver enzyme was observed [13–15]. In addition,* Tragopogon porrifolius *revealed effective antioxidant capability owing to its remarkable scavenging activity [13–15]. The objectives of the current work are to determine the AA, TPC, TFC, and anticancer activity of different extracts from* Tragopogon porrifolius* plant growing wild in Palestine. Antioxidants contents were assayed using FRAP, CUPRAC, DPPH, and ABTS colorimetric methods. TPC and TFC of the extracts were evaluated using Folin-Ciocalteu and aluminum chloride colorimetric methods, respectively. Anticancer activity was tested on HOS and KHOS osteosarcoma cell lines.

## 2. Materials and Methods

### 2.1. Plant Material

*Tragopogon porrifolius plant *was collected from the middle part of the West Bank, Palestine in February 2015. The plant was air-dried in dark at room temperature for two weeks, then milled to a powdered plant material, and then stored in fridge until extraction.

### 2.2. Chemicals and Reagents

2,4,6-Tripyridyl-S-triazine (TPTZ), hydrochloric acid 37% (w/w), sodium hydroxide, ferric chloride trihydrate, ferrous sulfate heptahydrate, potassium persulphate, sodium acetate, sodium carbonate, sodium nitrite, aluminum chloride, methanol, Folin-Ciocalteu reagent, Trolox (6-hydroxy-2,5,7,8-tetramethylchroman-2-carboxylic acid), gallic acid, cupper chloride, neocuproine, 99.9% ethanol, ammonium acetate, DPPH, methanol, ABTS (2,2-azino-di-(3-ethylbenzothialozine-sulphonic acid)), and potassium persulphate were all obtained from Sigma-Aldrich, Germany. All chemicals and reagents were of analytical grade. RPMI 1640, fetal bovine serum, antibiotics, and glutamine were purchased from Gibco.

FRAP reagent was prepared according to Benzie and Strain [16] by the addition of 2.5 mL of a 10 mM tripydyltriazine (TPTZ) solution in 40 mM HCl plus 2.5 mL of 20 mM FeCl_3_·6H_2_O and 25 mL of 0.3 M acetate buffer at pH 3.6. Acetate buffer (0.3 M) was prepared by dissolving 16.8 g of acetic acid and 0.8 g of sodium hydroxide in 1000 mL of distilled water.

### 2.3. Extraction of the Plant

Dry powder of plant material (five grams) was extracted separately with 50 mL of three extraction solvents (water, 80% ethanol, and 100% ethanol) in water bath at 37°C for three hours. The extracts were then filtered and the filtrate was stored at 4°C until used for analysis (TPC, TFC, and AA).

### 2.4. Measurement of Antioxidant Activity 

#### 2.4.1. FRAP Assay

The antioxidant activity of the extracts was determined using a modified method of the assay of ferric reducing/antioxidant power (FRAP) of Benzie and Strain [16]. Freshly prepared FRAP reagent (3.0 mL) was warmed at 37°C and mixed with 40 *μ*L of the extract and the reaction mixtures were later incubated at 37°C. Absorbance at 593 nm was read with reference to a reagent blank containing distilled water which was also incubated at 37°C for up to 1 hour instead of 4 min, which was the original time applied in FRAP assay. Aqueous solutions of known Fe^+2^ concentrations in the range of 2–5 mM were used for calibration, and results were expressed as mmol Fe^+2^/g.

#### 2.4.2. Cupric Reducing Antioxidant Power (CUPRAC Assay)

The cupric ion reducing antioxidant capacity of the extracts was determined according to the method of Apak et al. [17]. 100 *μ*L of sample extract was mixed with 1 mL each of 10 mM of cupper chloride solution, 7.5 mM of neocuproine alcoholic solution (99.9% ethanol), 1 M (pH 7.0) of ammonium acetate buffer solution, and 1 mL of distilled water to make final volume 4.1 mL. After 30 min, the absorbance was recorded at 450 nm against the reagent blank. Standard curve was prepared using different concentrations of Trolox. The results were expressed as *μ*mol Trolox/g.

#### 2.4.3. Free Radical-Scavenging Activity Using DPPH (DPPH Assay)

DPPH assay is based on the measurement of the scavenging ability of antioxidants towards the stable DPPH radical, and the procedure was done according to Brand-Williams et al. [18]. A 3.9 mL aliquot of a 0.0634 mM of DPPH solution in methanol (95%) was added to 100 *μ*L of each extract. The mixture was vortexed for 5–10 sec. The change in the absorbance of the sample extract was measured at 515 nm for 30 min till the absorbance reached a steady state. The percentage inhibition of DPPH of the test sample and known solutions of Trolox were calculated by the following formula:

(1) $$\begin{array}{c} \text{\% inhibition }=\frac{\left(A^{{}^{\circ}}-A\right)}{A^{{}^{\circ}}}\times 100, \end{array}$$

where *A*° is the absorbance of a solution of 100 *μ*L methanol 95% and 3.9 mL of DPPH at 515 nm and *A* is the absorbance of the sample extract at 515 nm. Methanol (95%) was used as a blank. Standard curve was prepared using different concentrations of Trolox. The results were expressed as *μ*mol Trolox/g.

#### 2.4.4. Free Radical-Scavenging Activity Using ABTS (ABTS Assay)

A modified procedure using ABTS (2,2-azino-di-(3-ethylbenzothialozine-sulphonic acid)) as described by Re et al. [19] was used. The ABTS stock solution (7 mM) was prepared through reaction of 7 mM ABTS and 2.45 mM of potassium persulphate as the oxidant agent. The working solution of ABTS^+∙^ was obtained by diluting the stock solution in 99.9% ethanol to give an absorption of 0.70 ± 0.02 at 734 nm. 200 *μ*L sample extract was added to 1800 *μ*L of ABTS^+∙^ solution and absorbance readings at 734 nm were taken at 30°C exactly 10 min after initial mixing (*A*). The percentage inhibition of ABTS^+∙^ of the test sample and known solutions of Trolox were calculated by the following formula.

% inhibition = ((*A*° − *A*)/*A*°) × 100, where *A*° is the absorbance of a solution of 200 *μ*L of distilled water and 1800 *μ*L of ABTS^+∙^ at 734 nm and *A* is the absorbance of the test sample at 734 nm. The calibration curve between % inhibition and known solutions of Trolox (50–1000 *μ*M) was then established. The radical-scavenging activity of the test samples was expressed as Trolox equivalent antioxidant capacity TEAC (*μ*mol Trolox/g sample).

### 2.5. Total Phenolic Content (Folin-Ciocalteu Assay)

Total phenolics were determined using Folin-Ciocalteu reagents [20].* Tragopogon porrifolius *plant extracts or gallic acid standard (40 *μ*L) was mixed with 1.8 mL of Folin-Ciocalteu reagent (prediluted 10-fold with distilled water) and allowed to stand at room temperature for 5 min, and then 1.2 mL of sodium bicarbonate (7.5%, w/v) was added to the mixture. After standing for 60 min at room temperature, absorbance was measured at 765 nm. Aqueous solutions of known gallic acid concentrations in the range of 10–500 mg/L were used for calibration. Results were expressed as mg gallic acid equivalents (GAE)/g sample.

### 2.6. Total Flavonoid Content

The determination of total flavonoids was performed according to the colorimetric assay of Kim et al. [21]. Distilled water (4 mL) was added to 1 mL of the extract in a test tube. Then, 0.3 mL of 5% sodium nitrite solution was added, followed by 0.3 mL of 10% aluminum chloride solution. Test tubes were incubated at ambient temperature for 5 minutes, and then 2 mL of 1 M sodium hydroxide was added to the mixture. Immediately, the volume of reaction mixture was made to 10 mL with distilled water. The mixture was thoroughly mixed using test tube shaker and the absorbance of the pink color developed was determined at 510 nm. Aqueous solutions of known catechin concentrations in the range of 50–100 mg/L were used for calibration and the results were expressed as mg catechin equivalents (CEQ)/g sample.

### 2.7. Cell Culture

HOS and KHOS human osteosarcoma cell lines were grown in RPMI 1640 medium supplemented 10% fetal bovine serum (FBS), 100 *μ*g/mL streptomycin, and 100 U/mL penicillin. Cells were maintained at 37°C in a humidified atmosphere of 5% CO_2_.

### 2.8. Anticancer Activity

The anticancer activity of the plant extract was evaluated over 3 days after treating osteosarcoma cell lines KHOS with either 2 mg/mL or 4 mg/mL of the crude plant extract. Additionally anticancer activity of the plant extract was evaluated over 2 days after treating osteosarcoma cell lines HOS with 4 mg/mL of the crude plant extract. Cell death was evaluated using trypan blue exclusion assay where cells were trypsinized, suspended, and counted using hemocytometer. Cell growth was measured by counting the cells over 3 days posttreatment.

### 2.9. Statistical Analyses

Three samples of* Tragopogon porrifolius* plant were independently analyzed and all of the determinations were carried out in triplicate. The results are expressed as means ± standard deviations.

## 3. Results and Discussion

### 3.1. Total Phenolic Contents (TPC)

TPC of* Tragopogon porrifolius *plant extracts using three different solvents is shown in Table 1. As it is obvious from this table, the extraction solvent has an effect on the TPC of the plant extracts where significant differences (*p* < 0.05) between the TPC of the three extracts are indicated by different small letters (a, b, and c). The highest TPC was found for the plant material when extracted with 80% ethanol (145.3 ± 3.1 mg/g), followed by plant material extracted with water (102.9 ± 3.9 mg/g) and finally with 100% ethanol (87.3 ± 1.8 mg/g). These results show that TPC were only 68% and 42% when the plant material was extracted by distilled water and 100% ethanol, respectively, as compared with the TPC extracted with 80% ethanol indicating the higher solubility of the phenolic compounds in 80% ethanol.

The results showed that* Tragopogon porrifolius *plant investigated in this study are richer with phenolic compounds (87.3 to 145.3 mg/g) than that from Turkey (from 63.4 to 68.9 mg/g caffeic acid) [4] or from Lebanon (37.0 mgGAE/g) [1].

### 3.2. Total Flavonoid Content (TFC)

The results of ferric chloride colorimetric test for determining flavonoids content are presented in Table 1. The same statistical analyses as for TPC were performed for total flavonoids content (TFC), and the results (Table 1) showed that significant differences between total flavonoids content of the plant materials extracted with the three solvents were obtained, where significant differences (*p* < 0.05) are indicated by small letters (a, b, and c). The highest TFC was found for the plant material when extracted with 80% ethanol (28.5 ± 0.2 mg/g) which is about two times significantly higher than that extracted with 100% ethanol (14.7 ± 0.3 mg/g) and the latter was significantly about three times higher than the TFC extracted with water (4.8 ± 0.3 mg/g). Different trend of solvent effect on TFC and TPC was obtained where the highest content of TPC and TFC was obtained when the plant was extracted with 80% ethanol, while the lowest was with 100% ethanol for TPC and for water for TFC; see Table 1. Apparently, mixed solvents of intermediate polarities (80% ethanol) are the most suitable extracting solvent for recovering the highest amounts of phenolic and flavonoid compounds which have both polar and nonpolar functional groups.

It was interesting to compare TFC of* Tragopogon porrifolius *plant analyzed in this study (range: 4.8–28.5 mg/g) with that from other countries (4 to 210 mg/g quercetin for* Tragopogon porrifolius* from Turkey [4] and 16.6 mg/g quercetin for* Tragopogon porrifolius* from Lebanon [1]).

### 3.3. Antioxidant Activity

AA accounts for the presence of efficient oxygen radical scavengers, such as phenolic compounds [22]. The antioxidant activity of phenolics is mainly due to their redox properties, which make them act as reducing agents, hydrogen donors, and singlet oxygen quenchers [22].

#### 3.3.1. Reducing Potential of Plant Extracts

*(1) FRAP Assay*. FRAP assay measures the reducing potential of an antioxidant reacting with a ferric tripyridyltriazine (Fe^3+^-TPTZ) complex and producing a colored ferrous tripyridyltriazine (Fe^2+^-TPTZ).

The antioxidant tests based on FRAP assay of* Tragopogon porrifolius *plant extracts using three different solvents are presented in Table 1 (expressed as mmol Fe^+2^/g of dry plant material). Statistical analyses showed that there are significant differences between FRAP values as a function of extraction solvent (Table 1), where significant differences (*p* < 0.05) are indicated by different small letters (a, b, and c).

Table 1 revealed that antioxidant activity (FRAP) of the* Tragopogon porrifolius *plant increased as the polarity of solvent changes (80% ethanol > water > 100% ethanol), where FRAP values were found to be about two and six times significantly higher when extracted with 80% ethanol compared to water and 100% ethanol, respectively.

The trend of extraction solvent on the FRAP values was found to be the same as for TPC but different from TFC. This suggests that there is a correlation between AA (expressed as FRAP) and TPC, reflecting the fact that total phenolics are the major determinant of AA.

As in the case of TPC and TFC, 80% ethanol gives higher amounts of AA (FRAP) compared with water as extraction solvent of* Tragopogon porrifolius*.

It is interesting to compare AA (FRAP) of Palestinian* Tragopogon porrifolius *with that from other countries. For example,* Tragopogon porrifolius *from Lebanon [1] was found to have 0.659 mmol Fe^+2^/g which is much lower than the FRAP value for* Tragopogon porrifolius *plant analyzed in this study (2.1–12.1 mmol Fe^+2^/g).

*(2) CUPRAC Assay*. Although FRAP antioxidant assay has been very popular among researchers, CUPRAC assay is a relatively new assay developed by Apak et al. [17]. It utilizes the copper(II)-neocuproine [Cu(II)-Nc] reagent as the chromogenic oxidizing agent and is based on the cupric reducing ability of reducing compounds to cuprous.

Table 1 shows the CUPRAC antioxidant activity (expressed as *μ*mole Trolox/g) of* Tragopogon porrifolius* plant extracts using three different solvents. Statistical analyses showed that there are significant differences between AA using the three extraction solvents, where significant differences (*p* < 0.05) are indicated by different small letters (a, b, and c).

Results showed that CUPRAC antioxidant activity of the* Tragopogon porrifolius *plant increased in the following order: 80% ethanol > water > 100% ethanol which is the same trend as FRAP antioxidant activity and TPC but different from TFC, which suggests that there is a correlation between CUPRAC AA and TPC.

#### 3.3.2. Free Radical-Scavenging Ability of Plant Extracts

*(1) DPPH Assay*. DPPH is a free radical compound and has been widely used to test the free radical-scavenging ability of various samples [23]. It is a stable free radical with a characteristic absorption at 517 nm that was used to study the radical-scavenging effects of extracts. As antioxidants donate protons to this radical, the absorption decreases. Antioxidants, on interaction with DPPH, transfer either an electron or hydrogen atom to DPPH, thus neutralizing its free radical character [24]. The color changed from purple to yellow and the absorbance at wavelength 517 nm decreased.

DPPH assay is based on the ability of the stable free radical 2,2-diphenyl-1-picrylhydrazyl to react with hydrogen donors including phenolics. The bleaching of DPPH solution increases linearly with increasing amount of extract in a given volume.

Table 1 shows the % inhibition of DPPH free radicals by the* Tragopogon porrifolius *plant extracted with the three solvents. Statistical analyses showed that there are significant differences between % inhibitions using the three extraction solvents, where significant differences (*p* < 0.05) are indicated by different small letters (a, b, and c); see Table 1.

Table 2 shows the % inhibition of DPPH at different concentrations of the crude extract (from 20 to 120 *μ*g/mL). This data shows that the extracts exhibited a dose dependent scavenging activity (a linear relationship between % of DPPH inhibition and concentration (*y* = 0.6995*x* − 0.9297, with *R* ^2^ of 0.9998), where *y* is the % of inhibition and *x* is the concentration). From this linear relationship, EC50 which is the concentration required to quench 50% of the DPPH free radicals was determined and was found to be 73 *μ*g/mL.

DPPH antioxidant activity of* Tragopogon porrifolius* plant extracts using three different solvents was expressed as *μ*mole Trolox/g (Table 1) and EC50 (Table 2). Statistical analyses showed that there are significant differences between AA using the three extraction solvents, where significant differences (*p* < 0.05) are indicated by different small letters (a, b, and c).

Results showed that DPPH antioxidant activity of the* Tragopogon porrifolius* plant increased in the following order: 80% ethanol > water > 100% ethanol which is the same trend as TPC, FRAP, and CUPRAC antioxidant activity.

*(2) ABTS Assay*. The ABTS assay measures the relative antioxidant ability of extracts to scavenge the radical-cation ABTS^+∙^ produced by the oxidation of 2,2′-azinobis-3-ethylbenzothiazoline-6-sulphonate.

Table 1 shows the % inhibition of ABTS free radicals by the plant extracted with the three solvents. Statistical analyses showed that there are significant differences between AA using the three extraction solvents, where significant differences (*p* < 0.05) are indicated by different small letters (a, b, and c); see Table 1.

Table 2 shows the % inhibition of ABTS at different concentrations of the crude extract (from 20 to 100 *μ*g/mL). This data shows that the extracts showed a dose dependent scavenging activity (a linear relationship between % of ABTS inhibition and concentration (*y* = 0.8105*x* + 1.35, with *R* ^2^ of 0.999), where *y* is the % of inhibition and *x* is the concentration). From this linear relationship, IC50 was determined and was found to be about 60.8 *μ*g/mL.

ABTS antioxidant activity of* Tragopogon porrifolius* plant extracts using three different solvents was expressed as *μ*mol Trolox/g (Table 1) and EC50 (Table 2). Statistical analyses showed that there are significant differences between AA using the three extraction solvents, where significant differences (*p* < 0.05) are indicated by different small letters (a, b, and c).

Results showed that ABTS antioxidant activity of the* Tragopogon porrifolius* plant increased in the following order: 80% ethanol > water > 100% ethanol which is the same trend as FRAP, CUPRAC, and DPPH antioxidant activities. Additionally this trend is the same as TPC but different from TFC, which suggests that there is a correlation between ABTS and TPC.

### 3.4. Anticancer Activity

**Figure 1:**
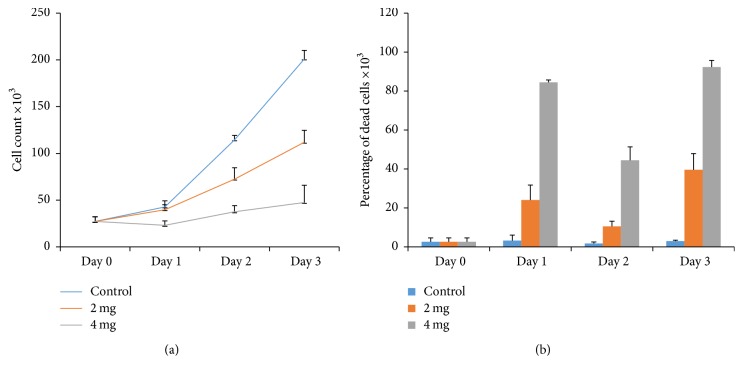
Cytotoxicity of* Tragopogon porrifolius *ethanolic extract on KHOS cancer cell lines. (a) Effect of the extract on cell growth after treating the cells with the indicated concentrations for the indicated time points. (b) Effect of the extract on cell viability after treating the cells with the indicated concentrations for the indicated time points. Error bars represent standard deviation calculated from three different experiments carried out in triplicate.

**Figure 2:**
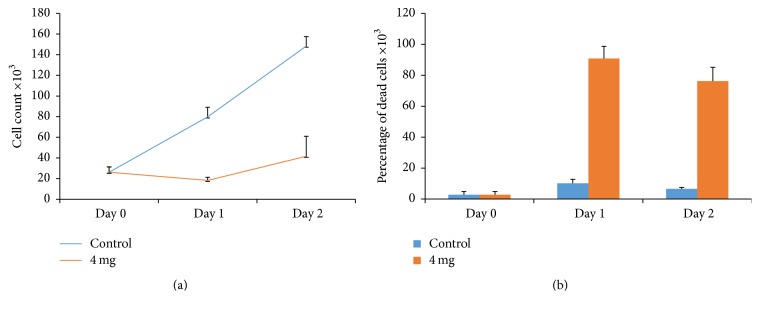
Cytotoxicity of* Tragopogon porrifolius *ethanolic extract on HOS cells. (a) Effect of the extract on cell growth after treating the cells with the indicated concentrations for the indicated time points. (b) Effect of the extract on cell viability after treating the cells with the indicated concentrations for the indicated time points. Error bars represent standard deviation calculated from three different experiments carried out in triplicate.

In order to test the anticancer activity of the crude extract of the Palestinian* Tragopogon porrifolius, *human osteosarcoma cells, HOS and KHOS, were treated with different concentrations of the extract (2 mg/mL and 4 mg/mL for KHOS and 4 mg/mL for HOS cells) and counted them and scored the percentage of dead cells. As shown in Figures 1(a) and 2(a), ethanolic extract decreased cell growth rate to less than 30% after three days for KHOS cells or two days for HOS cells. Moreover, the extract increased the percentage of dead cells by many folds as compared to control untreated cells (Figures 1(b) and 2(b)). Indeed our results are consistent with previous results that showed that* Tragopogon porrifolius* has a strong anticancer activity [1]. While it has been shown that* Tragopogon porrifolius* has a strong anticancer activity against both breast and colon cancer cell lines [1], we here succeeded to demonstrate an anticancer activity against two very aggressive osteosarcoma cell lines.

## 4. Conclusions

*Tragopogon porrifolius *plant is rich with phenolic compounds and flavonoids, and it has anticancer activities against two very aggressive osteosarcoma cancer cell lines (KHOS and HOS). Total phenolics and flavonoid contents as well as antioxidant activities are highest for plant extracted with 80% ethanol. Mixture of ethanol and water (80% ethanol) are the best solvent for extraction of phenolic and flavonoid compounds. There is a correlation between antioxidant activity and total phenolic content but not with total flavonoid content.* Tragopogon porrifolius *plant constitutes a natural source of potent antioxidants that may prevent many diseases and could be potentially used in food, cosmetics, and pharmaceutical industries.

## Figures and Tables

**Table 1:** Total phenolic content (TPC as mg gallic acid/g DW^*∗*^), total flavonoids contents (TFC as mg catechin/g DW), FRAP (mmol Fe^+2^/g DW), CUPRAC (*μ*mol Trolox/g DW), DPPH (*μ*mol Trolox/g DW), ABTS (*μ*mol Trolox/g DW), DPPH % inhibition, and ABTS % inhibition of *Tragopogon porrifolius *plant extracted with water, 80% ethanol, and 100% ethanol.

	TPC^*∗∗*^ (mg/g)	TFC (mg/g)	FRAP (mmol/g)	CUPRAC (*μ*mol/g)	DPPH (*μ*mol/g)	ABTS (*μ*mol/g)	DPPH % inhibition	ABTS % inhibition
Water	102.9^b^ ± 3.9	4.8^c^ ± 0.3	5.4^b^ ± 0.5	1945^b^ ± 33	226^b^ ± 7.1	44.1^b^ ± 2.2	77.3^b^ ± 1.3	80.2^b^ ± 1.4
Ethanol (80%)	145.3^a^ ± 3.1	28.5^a^ ± 0.2	12.1^a^ ± 0.8	4522^a^ ± 42	324^a^ ± 3.2	84.2^a^ ± 2.6	83.2^a^ ± 2.4	88.0^a^ ± 1.2
Ethanol (100%)	87.3^c^ ± 1.8	14.7^b^ ± 0.3	2.1^c^ ± 0.7	1232^c^ ± 41	117^c^ ± 2.5	29.4^c^ ± 1.2	70.5^c^ ± 1.6	78.3^c^ ± 2.0

^*∗*^DW: dry weight

^*∗∗*^Results are expressed as average of three samples of *T.* shoots. Different small letters within column indicate significant difference (*p* < 0.05, *n* = 3).

**Table 2:** % inhibition of DPPH and ABTS free radicals by different concentrations of *Tragopogon porrifolius *plant extract.

Concentration of DPPH (*μ*g/mL)	% inhibition of DPPH^*∗*,°^	Concentration of ABTS (*μ*g/mL)	% inhibition of ABTS^*∗*,°^
20	13.3 ± 0.5	20	16.9 ± 0.3
40	26.7 ± 1.2	40	34.1 ± 0.7
60	41.4 ± 1.0	60	50.3 ± 0.9
80	54.6 ± 1.4	80	67.2 ± 1.1
120	83.2 ± 2.1	100	81.4 ± 1.5

^*∗*^Results are expressed as average ± standard deviation of three samples.

°EC50 for DPPH and ABTS are 73 and 60.8 *μ*g/mL, respectively.

## References

[1] Tenkerian C., El-Sibai M., Daher C. F., Mroueh M. (2015). Hepatoprotective, antioxidant, and anticancer effects of the tragopogon porrifolius methanolic extract. *Evidence-Based Complementary and Alternative Medicine*.

[2] Formisano C., Rigano D., Senatore F., Bruno M., Rosselli S. (2010). Volatile constituents of the aerial parts of white salsify (*Tragopogon porrifolius* L., Asteraceae). *Natural Product Research*.

[3] Konopiński M. (2009). Influence of intercrop plants and varied tillage on yields and nutritional value of salsify (*Tragopogon porrifolius* L.) roots. *Acta Scientiarum Polonorum, Hortorum Cultus*.

[4] Özlem B. A., Gulcin S. C., Tülay Ç. (2013). Evaluation of antioxidant properties of some tragopogon species growing in Turkey. *Turkish Journal of Pharmaceutical Sciences*.

[5] Pham-Huy L. A., He H., Pham-Huy C. (2008). Free radicals, antioxidants in disease and health. *International Journal of Biomedical Science*.

[6] Servili M., Montedoro G. (2002). Contribution of phenolic compounds in virgin olive oil quality. *European Journal of Lipid Science and Technology*.

[7] Silva S., Gomes L., Leitão F., Coelho A. V., Boas L. V. (2006). Phenolic compounds and antioxidant activity of Olea europaea L. Fruits and leaves. *Food Science and Technology International*.

[8] Waris G., Ahsan H. (2006). Reactive oxygen species: role in the development of cancer and various chronic conditions. *Journal of Carcinogenesis*.

[9] Evans M. D., Dizdaroglu M., Cooke M. S. (2004). Oxidative DNA damage and disease: induction, repair and significance. *Mutation Research*.

[10] Prakash A., Rigelhof F., MIller E. http://www.medlabs.com/downloads/antiox_acti_.pdf.

[11] Mojarrab M., Khan Mohammadi A., Hosseinzadeh L., Siavash-Haghighi Z. M. (2014). Antioxidant activity and safety assessment of Tragopogon buphthalmoides hydroethanolic extract: acute and subchronic toxicities. *Research in Pharmaceutical Sciences*.

[12] Asadi-Samani M., Kafash-Farkhad N., Azimi N., Fasihi A., Alinia-Ahandani E., Rafieian-Kopaei M. (2015). Medicinal plants with hepatoprotective activity in Iranian folk medicine. *Asian Pacific Journal of Tropical Biomedicine*.

[13] Zeeni N., Daher C. F., Saab L., Mroueh M. (2014). *Tragopogon porrifolius* improves serum lipid profile and increases short-term satiety in rats. *Appetite*.

[14] Mroueh M., Daher C., El Sibai M., Tenkerian C. (2011). Antioxidant and hepatoprotective activity of *Tragopogon porrifolius* methanolic extract. *Planta Medica*.

[15] Govind P. (2011). Medicinal plants against liver diseases. *International Research Journal of Pharmacy*.

[16] Benzie I. F. F., Strain J. J. (1998). Ferric reducing/antioxidant power assay: direct measure of total antioxidant activity of biological fluids and modified version for simultaneous measurement of total antioxidant power and ascorbic acid concentration. *Methods in Enzymology*.

[17] Apak R., Güçlü K., Özyürek M., Çelik S. E. (2008). Mechanism of antioxidant capacity assays and the CUPRAC (cupric ion reducing antioxidant capacity) assay. *Microchimica Acta*.

[18] Brand-Williams W., Cuvelier M. E., Berset C. (1995). Use of a free radical method to evaluate antioxidant activity. *LWT—Food Science and Technology*.

[19] Re R., Pellegrini N., Proteggente A., Pannala A., Yang M., Rice-Evans C. (1999). Antioxidant activity applying an improved ABTS radical cation decolorization assay. *Free Radical Biology and Medicine*.

[20] Singleton V. L., Rossi J. A. (1965). Colorimetry of total phenolics with phosphomolybdic-phosphotungstic acid reagents. *American Journal of Enology and Viticulture*.

[21] Kim D.-O., Jeong S. W., Lee C. Y. (2003). Antioxidant capacity of phenolic phytochemicals from various cultivars of plums. *Food Chemistry*.

[22] Scalfi L., Fogliano V., Pentangelo A., Graziani G., Giordano I., Ritieni A. (2000). Antioxidant activity and general fruit characteristics in different ecotypes of Corbarini small tomatoes. *Journal of Agricultural and Food Chemistry*.

[23] Sakanaka S., Tachibana Y., Okada Y. (2005). Preparation and antioxidant properties of extracts of Japanese persimmon leaf tea (kakinoha-cha). *Food Chemistry*.

[24] Naik G. H., Priyadarsini K. I., Satav J. G. (2003). Comparative antioxidant activity of individual herbal components used in ayurvedic medicine. *Phytochemistry*.

